# AAPM BTSC Report 377: Physicist Brachytherapy Training in 2021—A survey of therapeutic medical physics residency program directors

**DOI:** 10.1002/acm2.13859

**Published:** 2023-01-18

**Authors:** Manik Aima, Samantha J. Simiele, Susan L. Richardson, Christopher S. Melhus

**Affiliations:** ^1^ Department of Radiation Oncology Stanford University Stanford California USA; ^2^ Department of Radiation Physics University of Texas MD Anderson Cancer Center Houston Texas USA; ^3^ Department of Radiation Oncology Swedish Medical Center Seattle Washington USA; ^4^ Department of Radiation Oncology Tufts University School of Medicine Boston Massachusetts USA

**Keywords:** brachytherapy, education, residency, survey, training

## Abstract

**Background:**

Brachytherapy (BT) was the first radiotherapeutic technique used to treat human disease and remains an essential modality in radiation oncology. A decline in the utilization of BT as a treatment modality has been observed and reported, which may impact training opportunities for medical physics residents. A survey of therapeutic medical physics residency program directors was performed as part of an assessment of the current state of BT training during residency.

**Methods:**

In March 2021, a survey consisting of 23 questions was designed by a working unit of the Brachytherapy Subcommittee of the American Association of Physicists in Medicine (AAPM) and approved for distribution by the Executive Committee of the AAPM. The survey was distributed to the directors of the Commission on Accreditation of Medical Physics Education Programs (CAMPEP)‐accredited therapeutic medical physics residency programs by the AAPM. The participant response was recorded anonymously in an online platform and then analyzed using MATLAB and Microsoft Excel software.

**Results:**

The survey was distributed to the program directors of 110 residency programs. Over the course of 6 weeks, 72 directors accessed the survey online, and 55 fully completed the survey. Individual responses from the directors (including partial submissions) were evaluated and analyzed. Nearly all participating programs (98%) utilize high dose rate BT treatments with 74% using low dose rate BT techniques. All programs treated gynecological sites using BT, and the next most common treatment sites were prostate (80%) and breast (53%). Overall, the residency program directors had a positive outlook toward BT as a radiotherapeutic treatment modality. Caseload and time limitations were identified as primary barriers to BT training by some programs.

**Conclusions:**

Based on the responses of the program directors, it was identified that the residency programs might benefit from additional resources such as virtual BT training, interinstitutional collaborations as well as resident fellowships. Programs might also benefit from additional guidance related to BT‐specific training requirements to help program directors attest Authorized Medical Physicist eligibility for graduating residents.

## 1 INTRODUCTION

Brachytherapy (BT) is an important radiotherapy modality that has been in use for various types of cancer treatments for over a century. Recently, there has been a decline in the utilization of BT as a treatment option.^1^, ^2^, ^3^, ^4^, ^5^ While this decline has clear implications for public health and cancer morbidity, there are related challenges inherent in training future generations of brachytherapists.^1^, ^2^, ^3^, ^4^, ^5^, ^6^ Declining utilization directly correlates with declining opportunities for medical physicists in training to attend BT procedures. The path to American Board of Radiology certification for a therapeutic medical physicist now requires a residency to prepare for providing clinical services. Residency programs are accredited by the Commission on Accreditation of Medical Physics Education Programs (CAMPEP) which is sponsored by several related professional societies, including the American Association of Physicists in Medicine (AAPM), the American College of Radiology (ACR), the American Association for Radiation Oncology (ASTRO), the Canadian Organization of Medical Physicists (COMP), and the Radiological Society of North America (RSNA). A set of essential guidelines provided by Report 249 of the AAPM regarding the training of medical physics residents includes a comprehensive list of BT‐related competencies (section 4.5.4).^7^ In this era of declining BT utilization, it is a valuable endeavor to assess the current state of BT‐related medical physics resident training to anticipate current and future needs as well as identify barriers to resident training.

The Brachytherapy Subcommittee (BTSC) of the Therapy Physics Committee (TPC) under the Science Council of the AAPM pursued an investigation into the current state of residency training. A working unit was formed, comprising of two senior subcommittee members and two junior members. The unit proposed to evaluate the state of BT training through surveys of the trainers (i.e., residency program directors) and the trainees (i.e., residents). This work presents the results of a survey that was distributed to the CAMPEP‐accredited residency program directors, with a goal to evaluate medical physics resident training in the areas of high dose rate (HDR), low dose rate (LDR), radiopharmaceutical, and electronic BT. Through this survey, the authors collected information about the various BT modalities used at different training programs: types and frequency of cases, distribution and frequency of treatment sites, typical workload/caseload per resident, and availability of adequate and diverse learning opportunities involving residents. Information regarding attestation of the training and experience requirements documented on the Nuclear Regulatory Commission (NRC) form 313A^8^ was also gathered. This attestation may have implications for a newly employed physicist's ability to serve as an Authorized Medical Physicist (AMP), i.e., be listed on an institution's radioactive materials license.

## 2 MATERIALS AND METHODS

### 2.1 Survey design

The survey consisted of 23 questions of four types including multiple choice, select all that apply, five‐point scale, and free response. The questions were designed to collect information regarding six areas: (1) the types of modalities, radioactive sources, treatment sites, and dose calculation algorithms utilized at each program; (2) the average annual patient‐ and fraction‐based caseload at each program as well as the length of time residents spend training in BT; (3) AMP eligibility requirements as they relate to the training received during a residency program; (4) program directors’ views on BT as a treatment modality; (5) program directors’ views on the BT training received by residents both generally as well as specific to their own program; and (6) a free response question to identify additional thematic information not directly asked during the survey. A copy of the survey questions is provided in the Appendix. Marcrom et al.^6^ designed a similar survey for radiation oncology residents, the results of which were published in 2018.

### 2.2 Approval process

The survey content was thoroughly vetted by the AAPM. The survey was first reviewed and approved by the BTSC before undergoing review by the TPC. The survey was then distributed to both the Professional and Education Councils for comments and approval. Following consideration and incorporation of feedback, the survey was presented to the Executive Committee (ExCom) of the AAPM by the Education Council. The survey was approved for distribution to the residency program directors by ExCom.

### 2.3 Survey distribution

A link to the survey was distributed among the program directors according to the CAMPEP‐accredited therapy residency program list (March 2021).^9^ This list included 110 different programs. The survey invitation was sent by AAPM headquarters staff, and a response was requested within 6 weeks. Reminder emails were sent at the completion of weeks 3 and 5 as well as a final reminder that was sent on the day of the survey deadline.

### 2.4 Analysis

Numerical results were analyzed using summary statistics with Microsoft Excel^10^ and MATLAB^11^ software programs. Responses from participants that consisted of free‐form text were collated using identification of keywords and authors’ interpretation of the comments. Common pertinent themes were identified based on the comments and are summarized in the Results section.

## 3 RESULTS

### 3.1 Survey response rate

Seventy‐two program directors responded to the survey out of 110 survey recipients. Fifty‐five program directors completed the full survey for an overall response rate of 65% and a completion rate of 50%. Surveys were considered “completed” if respondents electronically submitted the survey, even though they may not have answered all questions. All survey answers were included in the analysis independent of whether a program director completed the survey in full or in part. The average time spent on the survey by all respondents was 27 min. The average time required to complete the survey was slightly greater at 33 min.

### 3.2 Summary of program director reported data

#### 3.2.1 Modalities and treatment sites at programs

Program directors were asked about the treatment sites, source types, treatment modalities, caseload for each modality, dose calculation algorithms, imaging modalities, and use of therapeutic radionuclides at their respective institutions. This information allowed evaluation of the breadth of procedures in which residents receive BT‐related training.

**FIGURE 1:**
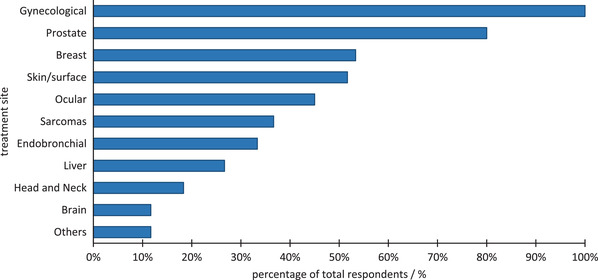
The institutional BT treatment site distribution reported by the program directors

The distribution of the utilization of BT for various treatment sites is presented in Figure 1. All program directors (100%) out of a total of 60 who answered this question reported treating gynecological (GYN) sites using BT, followed by prostate (80%) and breast (53%). Skin/surface, ocular, sarcomas, endobronchial, liver, head and neck, as well as brain cancer treatments using BT were also reported.

**FIGURE 2:**
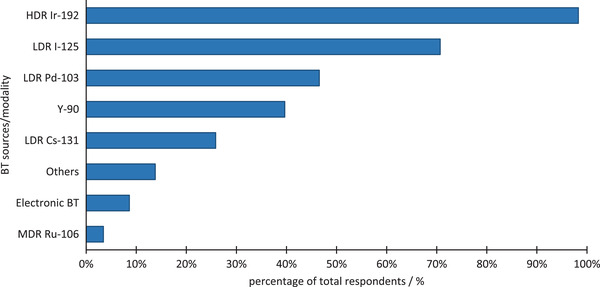
The list of BT sources used by various programs. MDR, medium dose rate

A variety of BT sources are used by the survey participants for treatments. Individual respondents could select multiple sources for this question. The distribution of sources utilized for BT treatments by the programs is presented in Figure 2.

An overwhelming majority of respondents (out of a total of 58) use HDR Ir‐192‐based BT (98%). LDR BT was utilized by 74% of the respondents including I‐125 (71%) and Pd‐103 (47%).

**FIGURE 3:**
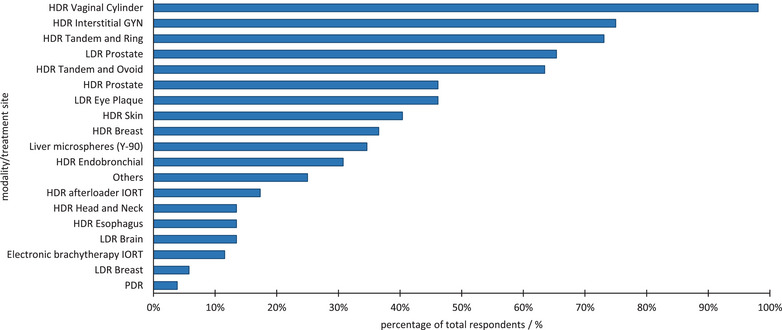
Reported distribution of the various BT modalities and treatment sites utilized by the programs. IORT, intraoperative radiotherapy

**FIGURE 4:**
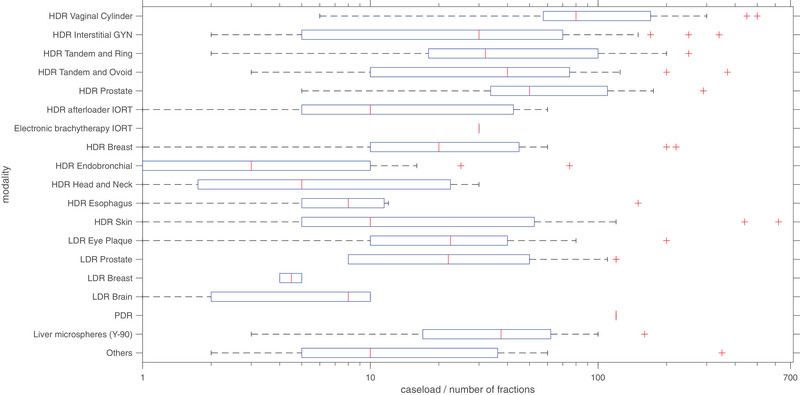
Annual caseload reported by program directors for various BT modalities. IORT, intraoperative radiotherapy. *Note*: Abscissa is in logarithmic scale. The box plot illustration comprises of the red mark in the center indicating the median, with the top and the bottom edges indicating the 75th and the 25th percentiles of the data, respectively. The whiskers extend to the extreme data points that are not designated as outliers, and the data outliers are illustrated with the “+” marker symbol^11^

The results of the reported BT technique and treatment site distribution are presented in Figures 3 and 4. Fifty‐one out of 52 respondents to this question (98%) utilize HDR vaginal cylinder treatments, followed by 75% of the respondents using HDR interstitial GYN treatments, and 73% using HDR tandem‐and‐ring treatments. LDR prostate treatments and HDR tandem‐and‐ovoid treatments are performed by approximately two‐thirds of the programs. The distribution of other treatment sites presented in Figure 3 contrasts the utilization of BT among different programs for sites such as ocular, skin, breast, liver, and endobronchial and showcases the variety of clinical applications in which BT is utilized.

In terms of caseload, as seen in Figure 4, the use of BT for treatment of GYN cancers was most common. Many programs specialized or had exceedingly large numbers of fractions compared with the average for certain procedures such as skin, liver, and ocular BT.

Most (98%) programs reported using the AAPM TG43 formalism^12^ as their BT dose calculation algorithm. Fifteen percent of the programs reported using model‐based dose calculation algorithms (MBDCAs) as well, with one program solely using MBDCAs. Finally, 4% of the programs reported using a TG43 hybrid calculation approach.

All but one program reported using computed tomography (CT) as an imaging modality for BT. Close to 80% of the programs reported using magnetic resonance imaging (79.6%) or ultrasound (77.8%) while performing BT procedures. Other imaging modalities frequently used included fluoroscopy (24.1%), digital radiography (18.5%), single‐photon emission computerized tomography (11.1%), and others (5.6%). Other modalities included PET, DYNA CT, and linac‐based kV imaging. Seventy‐four percent of the programs used three or more modalities for BT procedures.

Approximately half (48%) of the programs reported residents receiving training in the use of therapeutic radionuclides. The list of radionuclides used is presented in Table 1 along with the percentage of programs using each specific radionuclide. The calculated percentages are normalized to the total number of programs who reported training their residents in the use of therapeutic radionuclides.

**TABLE 1:** Percentage of programs treating with specific radiopharmaceuticals

Radionuclides	No. of Programs	Overall percentage radionuclide use (%)
Cs‐131	1	3.8
Cs‐137	1	3.8
I‐125	1	3.8
I‐131	5	19.2
Lu‐177	5	19.2
Ra‐223	14	53.8
Sm‐153	1	3.8
Sr‐89	2	7.7
Y‐90	17	65.4

#### 3.2.2 BT training details

Program directors were asked about the annual BT caseload at their institution in terms of patients treated as well as the number of fractions. Additionally, data were collected about the length of time residents spend training in BT in terms of months on a BT rotation as well as the number of training hours received.

Twenty‐two percent of programs reported an annual caseload between 101 and 150 BT patients each year, with 21% of programs treating more than 300 patients each year using BT. Only three programs reported 50 or fewer cases per year.

The plurality (31%) of programs reported performing more than 500 BT fractions each year with 21% of programs performing between 201 and 300 fractions each year. Twenty‐five percent of the directors reported 200 or fewer fractions treated per year (and no programs reported 50 or less).

Most programs (66%) require residents to spend 3–4 months on a BT rotation. Five percent of programs reported not having a dedicated BT rotation. All programs that offer a dedicated BT rotation reported residents spending no fewer than 2 months training in BT. Fourteen percent of the programs reported residents spending more than 5 months for BT‐related training. The plurality (44%) of programs reported residents receiving between 300 and 500 hours of BT‐specific training.

Fifty‐seven percent of the program directors reported attesting to AMP eligibility for their graduating residents, while 43% do not. When program directors were asked if their program would benefit from more structured guidelines for the number of cases required for a physicist to become an AMP, 42.6% of program directors responded in the affirmative and 14.8% replied in the negative. The remaining 42.6% were unsure if their program would benefit from more detailed guidelines.

#### 3.2.3 Program directors’ view of BT as a modality

Program directors were asked about the importance of BT as a treatment modality, whether they thought BT utilization will increase in the future, as well as their thoughts on the adequacy of the status of BT training.

When asked about the importance of BT as a treatment modality in radiation oncology, 86% of program directors believed it was essential, 3% were neutral, and 11% were ambivalent.

When asked about the future of BT utilization for treating cancer, most respondents (57%) believed there will be some degree of increase, while 39% of the respondents believed there would be no change, and 4% believed there will be a decrease in utilization.

None of the program directors reported believing that the BT training was optional. A third of the directors believed that the training was adequate, 30% believed it requires some expansion, and 37.5% of the directors believed significant expansion was needed to the BT training.

#### 3.2.4 Program directors’ view of resident training

Program directors were asked to provide their opinions on the preparedness of their residents at the end of the residency training program, the degree of support from radiation oncology colleagues in allowing medical physics residents to engage in BT procedures, and the level of independence achieved by residents for different BT tasks. Additionally, directors were asked if a BT fellowship would be beneficial to their residents, what the greatest hurdles are in providing BT training, and what resources would be needed to overcome these hurdles. Finally, programs were asked what percentage of their residents practice BT in their first postresidency position.

**FIGURE 5:**
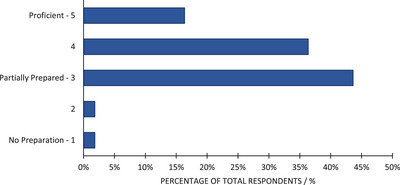
Perceived level of preparedness of residency program graduates to independently practice BT

The plurality (43.6%) of program directors believed CAMPEP‐accredited programs partially prepare residents to independently practice BT in their first postresidency position, with 36.4% of the directors perceiving their residents as more than partially prepared but not proficient. The results are shown in Figure 5, with 16.4% of the directors believing residents are proficient in BT after finishing their residency program. Only two programs (3.6%) believed that their residents are underprepared.

When asked if radiation oncologists are generally supportive of medical physics residents being involved in BT procedures, most (59%) program directors believed radiation oncologists were encouraging, while 28.6%, 10.7%, and 1.8% of directors felt radiation oncologists were supportive, neutral, or unsupportive, respectively.

**FIGURE 6:**
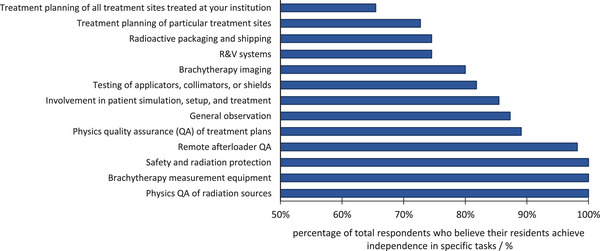
Percentage of respondents who believe their residents achieve independence in specific tasks related to BT. R&V, record and verify

Figure 6 presents the distribution of program directors who believed their residents achieve independence in various clinical BT‐related tasks. All program directors believed their residents achieved independence in three areas: (1) safety and radiation protection, (2) BT measurement equipment, and (3) physics quality assurance (QA) of radiation sources. Treatment planning was the area in which the fewest number of programs believed residents achieve independence. Only 65.5% of program directors believed their residents achieve independence in treatment planning for all sites treated at their program, and only 72.7% of directors believed independence is achieved by residents in planning of specific treatment sites.

Most (87.3%) respondents believed their residents could benefit (34.6%) or could maybe benefit (52.7%) from a BT fellowship. Only 12.8% of program directors believed their residents would not benefit from such a fellowship.

Approximately a third of program directors (32.1%) were unsure if graduates of their program practiced BT in their first postresidency position. The results are provided in Table 2.

**TABLE 2:** Reported distribution of residents practicing BT as part of their first employment post‐residency

Percentage of residents who practice brachytherapy in their first position					
Unknown	0%–20%	20%–40%	40%–60%	60%–80%	80%–100%
32.1%	7.5%	18.9%	13.2%	17.0%	11.3%

#### 3.2.5 Free response: Identification of barriers to adequate resident training

The greatest hurdle identified by residency directors regarding resident training was caseload. This includes the lack of treatment options and/or variety of case types that are handled at various programs. The second largest reported barrier was regarding time constraints including both lack of time for daily activities and on a larger scale such as the residency duration itself. Some procedures and modalities would not occur during a trainee's BT rotation, which correlates with a lack of caseload. A resident's limited time commitment while balancing other clinical tasks was also noted.

Many respondents recognized they could not affect a change in patient‐load constraints and focused on alternatives such as virtual resources for resident training. This included access to a database of treatment scans and plans, online learning/lecture series, and simulation labs. Others suggested longer residency training. A common theme was a need for higher levels of support for BT procedures ranging from physician acknowledgment of physicist necessity to more space and patience from the staff.

Some centers reported no concerns with caseload or time constraints. Interestingly, centers with large patient volumes (>300 cases per year) were equally likely to report concerns with time constraints and caseloads, indicating even large patient numbers alone are not a reliable solution for case and modality diversity.

Several respondents expressed apathy or lack of ability to change their training opportunities as barriers to BT training. While this may reflect the inability of an individual to change practice patterns at their institution, some expressed the feeling that BT use was declining following the patterns of care described earlier.^1^, ^2^, ^3^, ^4^, ^5^, ^6^ Collaboration with other institutions, fellowship programs, and AAPM‐sponsored cross‐training were ideas that would allow more hands‐on opportunities.

Several respondents indicated a desire to have detailed guidelines and caseloads like medical radiation oncologists when attesting to AMP license requirements. Another recurring theme was that of needed collaboration between various programs to afford residents with exposure to diverse caseloads, particularly those involving newer modalities and radiopharmaceutical administration.

## 4 DISCUSSION

One method to display a data‐driven look at strengths and weaknesses of a particular system is to perform a Strength, Weakness, Opportunities, and Threats (SWOT) analysis. In Table 3, relevant survey questions and answers have been assigned an appropriate quadrant for quick overview and review. A topic was assigned to internal factors if residency directors and/or institutions could potentially influence or change the topic. A topic was assigned to external factors if outside institutions and organizations or cancer care in general were responsible.

**TABLE 3:** SWOT analysis of therapeutic medical physics resident BT training

Internal factors		Internal factors	
Strengths (+)	Reference	Weaknesses (–)	Reference
Wide variety of sites and modalities are offered at most institutions	Q1	Some institutions reported not having a brachytherapy specific rotation	Q4
Almost half of institutions report at least 200 brachytherapy fractions per year	Q2	Only half of institutions provide training in radiopharmaceuticals	Q10
Radiation oncologists are supportive of medical physics residents	Q15	A majority of program directors believe expansion is needed to the current training received by residents in brachytherapy	Q13
A large majority of program directors reported brachytherapy as an essential modality	Q11	A large percentage of program directors find their residents only partially prepared or not prepared to practice brachytherapy independently upon residency completion	Q14

External factors		External factors	
Opportunities (+)	Reference	Threats (–)	Reference
Residents could potentially benefit from a brachytherapy fellowship	Q17	Program directors believe there will be no change or decrease in usage of brachytherapy in the future	Q12
More virtual resources and cross‐collaboration with other institutions could provide training when opportunities are missing	Q19	Caseloads and variety of cases for brachytherapy patients is dwindling	Q18
Many program directors attest to AMP eligibility, attestation rates should be higher with clear requirements and case load numbers	Q21 & Q22	Residents have time constraints which impede brachytherapy training	Q18
Update of Report 249 to correlate with modern brachytherapy clinics	Q23	Lack of mentorship/fellowship opportunities	Q19

AAPM Report 249 characterizes the guidelines and essential curriculum for a residency in medical physics.^7^ Published in 2013, it updates prior reports written in 2006 and 1992.^7^ The report is now nearly 10 years old and may not reflect the current state of professional practice and patterns of care. The stated requirements for medical physicists are generally topical and do not require a specific number of cases. In comparison, training radiation oncologists are required to accumulate and document a minimum number of cases in various body sites, using various modalities and techniques.^8^ In order to understand how well contemporary medical physics residencies can address the requirements of the Report 249, requirements were evaluated and grouped by topical area. These topical areas are discussed in this section.

According to AAPM report 249, the trainees should demonstrate an understanding in a variety of treatment sites including GYN, prostate, and soft tissue sarcomas, as well as optional sites such as the eye, and the liver. Training modalities included HDR, LDR, interstitial BT as well as optional modalities such as pulsed dose rate (PDR), electronic BT, and Y‐90 microsphere applications. Based on the information presented from responses to survey questions 1 and 6, the authors found that approximately 100% of centers treated GYN, 80% prostate, 45% ocular, 35% sarcoma, and only 12% were involved with electronic BT. About 40% of the programs reported using Y‐90, and 5% PDR.

The greatest barrier to adequate resident training identified by the residency directors was caseload. This includes the lack of modalities and/or variety of case types that are handled at various programs. Programs that treated fewer than 150 patients a year were more than twice as likely to report issues than programs who treated greater than 150 patients per year. This result reaffirms the finding of Marcrom et al., which was reported for physician residents.^6^ The authors reported “caseload was the greatest perceived barrier in BT training, with confidence correlated with case volume.^6^”One solution to address the lack of caseload may be the adoption of simulation trainings that use anthropomorphic phantoms as described by several authors.^13^, ^14^, ^15^

On a larger scale, the disparity of time allotted for BT training varied from no formal training to more than 5 months with the average time being 3 months. Time constraints on the treatment team were also indicated by the respondents, for example, in having limited time to train the residents in these modalities, which may be partially due to the urgent nature and short timeframe of BT procedures.

Regarding treatment planning and medical dosimetry, trainees are commonly required to perform a decay calculation and calculate the total dose delivered by a temporary and permanent implant as outlined by Report 249 recommendations. These are general and can be performed in a classroom setting. For specific plans, the report requires a treatment plan for a vaginal cylinder applicator, and inspection of Figure 3 shows that this can readily be met by 100% of responding program directors. Considering a treatment plan utilizing a cervical applicator, Figure 3 shows that approximately 75% and 65% of programs can provide the service for a tandem‐and‐ring and a tandem‐and‐ovoid, respectively. While clinics may offer one (or both), it is not clear that all programs can easily provide this service using internal resources. Finally, for an optional dose calculation requirement for a microsphere BT treatment, Figure 3 shows that fewer than 40% of programs report practicing microsphere therapy. Similarly, ocular BT is included as an optional training topic, although it is also only utilized at approximately 45% of responding programs. In summary, most programs will not be able to offer the optional training experiences without external assistance.

For the radiation safety requirements listed in Report 249, most requirements can be performed in the classroom or laboratory setting. These include performing source receipt procedures, source management activities, room surveys, regular inventories, leak checks for sealed sources, shielding calculations, and shielding surveys. Figure 6 presents the distribution of respondents who believe their residents achieve independence in some of these tasks.

For source assays or calibrations, Report 249 requires discussion and performance of an assay for sealed sources. If possible, it also recommends assays for unsealed sources; however, as noted above, most programs will not be able to offer this experience to their trainees. Finally, the trainee is expected to discuss and perform verification of source strength and reconcile their measurements with the vendor's certificate.

Finally, Report 249 has several items related to QA activities for trainees. These include demonstrating an understanding of and participating in periodic spot checks, safety procedures, and source exchange QA, including source calibration. In addition, there is a requirement to understand the comprehensive periodic QA of a remote afterloader. Considering that nearly all programs offer HDR Ir‐192 treatments, most of these items should be readily achievable for most clinics. Additional QA training topics include secondary dose calculations, treatment planning system QA, and acceptance testing for metrology equipment. These items are generally applicable and should be achievable at most training programs.

Our survey indicated program directors are divided on whether to attest to AMP eligibility via NRC form 313A for residents at the completion of residency training. This could pose a challenge for graduates seeking employment at institutions offering BT services. This study included program directors reporting the amount of time residents spend receiving BT training in units of months, with some residents receiving as few as 2 months, while others received more than 5 months. Of note, form 313A for AMPs requires an accounting of training duration in units of years but does not clarify if the training must be specific to BT or general training inclusive of BT.^8^, ^16^ Uncertainty regarding whether graduates of medical physics training programs meet AMP eligibility requirements is evident from the program directors’ responses. Most respondents, that is, 87% of the program directors who stated they do not attest to AMP eligibility and 84% of the program directors who stated they do attest to AMP eligibility, reported that their program would benefit from having more structured guidelines for attesting to AMP eligibility. Only 14.9% of program directors stated their program would not benefit from additional guidance regarding completion of NRC form 313A.

There are differences in the requirements of form 313A for authorized user radiation oncologists (AU) compared with AMPs.^8^, ^16^ Most notably, the training and education requirements for AUs are measured in hundreds of hours, while training and education requirements for AMPs are measured in years. The duration of residency training for AUs is generally twice as long as that of AMPs, making it easier to assure compliance with the AU requirements. In addition, the AU eligibility criteria directly references the Accreditation Council for Graduate Medical Education residency training requirements, which include specification of a minimum number of clinical cases. In contrast, the CAMPEP requirements for BT training leave caseload requirements to the discretion of the local program director. Thus, there is the potential for more variability in AMP training compared with AU training.

Data collected during this study could be used to inform the types of BT treatments and the number of cases per treatment type that training centers could accommodate based on typical caseloads. Additional effort would be needed to identify ways for residents to receive additional training experiences if their program did not offer the minimum requirements. Potential solutions include virtual offerings, simulations,^17^ and workshops. Medical physicists should review the current AMP requirements and determine whether residency training programs should be designed to give program directors confidence to attest to form 313A (or equivalent) for BT. Alternatively, the medical physics community may petition the NRC to update or modify AMP criteria to better reflect modern medical physicist training and preparation. Addressing this challenge is important, as it represents a potential barrier for physicists to practice BT.

This work was limited to surveying CAMPEP‐accredited residency programs. The data as well as accompanying analysis about BT as a treatment option and its availability are thus representative of programs of a similar nature and may not be representative of all residency programs. Additionally, this survey reflects opinions and training activities reported by medical physics residency program directors during the spring of 2021, and the results may have been influenced by the SARS CoV‐2 pandemic‐related implications.

## 5 CONCLUSIONS

A professional society‐driven survey of medical physics residency program directors was performed to glean insight into the current state of training specific to the practice of BT. Based on the responses of the program directors, it was identified that residency programs might benefit from additional resources to support training such as simulations, fellowships, and external rotations in an era of declining utilization. In addition, responses demonstrated that, specific to the practice of BT, there was little commonality between programs due to the variety of sites, techniques, and modalities. This challenges uniformity of practice and training in accredited programs. Finally, program directors might benefit from additional guidance related to attesting AMP eligibility for graduating residents.

## AUTHOR CONTRIBUTIONS

All authors worked on all the aspects of the manuscript. Manik Aima and Samantha J. Simiele contributed equally to the publication and should be considered joint first authors. Two authors acted as more senior authors and two as more junior in a mentorship arrangement. The survey design, results, and written summary were all contributed to by all authors.

## CONFLICT OF INTEREST

The Chair of the BTSC Unit 72 has reviewed the required Conflict of Interest statement on file for each member of BTSC Unit 72 and determined that disclosure of potential Conflicts of Interest is an adequate management plan. The members of Unit 72 attest that they have no potential Conflicts of Interest related to the subject matter or materials presented in this document.

## 1 PROGRAM DIRECTORS’ SURVEY QUESTIONS

1. What are the sites treated using brachytherapy at your institution? Select all that apply.
  - Breast
  - Gynecological (GYN)
  - Prostate
  - Sarcomas
  - Skin/Surface
  - Endobronchial
  - Ocular
  - Head and Neck
  - Brain
  - Liver
  - Others
2. What is the approximate annual brachytherapy patient‐based caseload at your institution?
  - 1–25
  - 26–50
  - 51–75
  - 76–100
  - 101–150
  - 151–200
  - 201–300
  - More than 300
3. What is the approximate annual brachytherapy fraction‐based caseload at your institution?
  - 1–50
  - 51–100
  - 101–150
  - 151–200
  - 201–300
  - 301–400
  - 401–500
  - More than 500
4. What is the average length of time residents are on a brachytherapy rotation?
  - 0–1 month
  - 1 month
  - 2 months
  - 3 months
  - 4 months
  - 5 months
  - More than 5 months
  - Our program does not have a dedicated brachytherapy rotation
5. What is the minimum number of hours in training in brachytherapy do you believe your residents receive?
  - 1–100
  - 101–200
  - 201–300
  - 301–400
  - 401–500
  - 501–600
  - More than 600
6. What type of sources are used at your facility? HDR, MDR, LDR, and PDR refer to high dose rate, medium dose rate, low dose rate, and pulsed dose rate brachytherapy respectively. Check all that apply.
  - HDR ^192^Ir
  - MDR ^106^Ru
  - LDR ^103^Pd
  - LDR ^131^Cs
  - LDR ^125^I
  - ^90^Y
  - Electronic brachytherapy
  - Others
7. What modalities are used? Please mark all that apply. Also, if applicable, provide an approximate estimate of the caseload (number of fractions) treated annually for that modality.
  - HDR vaginal cylinder
  - HDR interstitial GYN
  - HDR tandem and ring
  - HDR tandem and ovoid
  - HDR prostate
  - HDR remote afterloader intraoperative radiotherapy (IORT)
  - HDR electronic brachytherapy IORT
  - HDR breast
  - HDR endobronchial
  - HDR head and neck
  - HDR esophagus
  - HDR skin
  - LDR eye plaque
  - LDR prostate
  - LDR breast
  - LDR brain
  - PDR
  - Non‐invasive, image‐guided, breast brachytherapy
  - Liver microspheres (^90^Y)
  - Others
8. What type of dose calculation algorithms are used clinically at your institution? Select all that apply.
  - AAPM TG‐43
  - Model‐based dose calculation algorithm (MBDCA)
  - TG‐43 hybrid
  - Others
9. Which imaging modalities are used for brachytherapy at your institution? Select all apply.
  - Digital radiography
  - Ultrasound
  - Computed Tomography (CT)
  - Magnetic Resonance Imaging (MRI)
  - Fluoroscopy
  - Single‐photon emission computerized tomography (SPECT)
  - Others
10. Does resident training include therapeutic radionuclide treatments at your institution?
  - Yes
  - No
11. Please rank the following statement on the scale of (1) not essential – (3) neutral – (5) essential: Brachytherapy is an essential modality in radiation therapy.
  - 1
  - 2
  - 3
  - 4
  - 5
12. Please rank the following statement on the scale of (1) not utilized – (3) no change – (5) expanded utilization: Brachytherapy will be an important modality in radiation therapy in the future.
  - 1
  - 2
  - 3
  - 4
  - 5
13. Please rank the following statement on the scale of (1) optional elective – (3) adequate focus – (5) needs significant expansion: Training in brachytherapy is a necessary component in medical physics residency programs.
  - 1
  - 2
  - 3
  - 4
  - 5
14. Please rank the following statement on the scale of (1) no preparation – (3) partially prepared – (5) proficient: CAMPEP‐accredited medical physicist residency programs sufficiently prepare physicists to be independent brachytherapy physicists in their first position/employment
  - 1
  - 2
  - 3
  - 4
  - 5
15. Please rank the following statement on the scale of (1) discouraging – (3) neutral – (5) encouraging: Physician radiation oncologists are generally supportive of medical physics residents being involved in brachytherapy procedures.
  - 1
  - 2
  - 3
  - 4
  - 5
16. What level of independence do residents achieve during their training? Check all that apply.
  - General observation
  - Treatment planning of particular treatment sites
  - Treatment planning of all treatment sites treated at your institution
  - Physics quality assurance (QA) of treatment plans
  - Involvement in patient simulation, setup, and treatment
  - Safety and radiation protection
  - Radioactive packaging and shipping
  - Brachytherapy measurement equipment
  - Physics QA of radiation sources
  - Brachytherapy imaging
  - R&V systems
  - Remote afterloader QA
  - Testing of applicators, collimators, or shields
17. Do you believe your residents could benefit from a brachytherapy fellowship training program through the American Association of Physicists in Medicine such as the one offered by the American Brachytherapy Society for physicians?
  - Yes
  - No
  - Maybe
18. What do you think is the greatest hurdle in providing brachytherapy training to your residents?
19. What resource(s) would your program require to overcome your answer to the previous question?
20. What percentage of your residency graduates practice brachytherapy in their first post‐residency position?
21. Do you attest to your residents being Authorized Medical Physicist (AMP) eligible at the time of the residency program completion?
  - Yes
  - No
22. Would your program benefit from having more structured guidelines for the number of cases required for a physicist to become an AMP? Currently, the number of cases for a radiation oncology resident is well defined in 10CFR35, but the requirements are less structured for medical physicists.
  - Yes
  - No
  - Maybe
23. Is there additional information that you would like to provide?
